# Suitableness of SLM Manufactured Turbine Blade for Aerodynamical Tests

**DOI:** 10.3390/ma16072866

**Published:** 2023-04-04

**Authors:** Janusz Telega, Piotr Kaczynski, Małgorzata A. Śmiałek, Piotr Pawlowski, Ryszard Szwaba

**Affiliations:** 1 Institute of Fluid Flow Machinery Polish Academy of Sciences (IMP PAN), Fiszera 14, 80-231 Gdansk, Poland; 2 Faculty of Mechanical Engineering and Ship Technology, Institute of Naval Architecture and Ocean Engineering, Gdansk University of Technology, Narutowicza 11/12, 80-233 Gdansk, Poland; 3 Institute of Fundamental Technological Research Polish Academy of Sciences (IPPT PAN), Pawinskiego 5B, 02-106 Warsaw, Poland

**Keywords:** rapid prototyping, Selective Laser Melting SLM, Direct Metal Laser Sintering (DLMS) technology, additive manufacturing, compressor blade, experimental aerodynamics

## Abstract

This paper describes some insights on applicability of a Selective Laser Melting and Direct Metal Laser Sintering technology-manufactured turbine blade models for aerodynamic tests in a wind tunnel. The principal idea behind this research was to assess the possibilities of using ‘raw’ DLMS printed turbine blade models for gas-flow experiments. The actual blade, manufactured using the DLMS technology, is assessed in terms of surface quality (roughness), geometrical shape and size (outline), quality of counterbores and quality of small diameter holes. The results are evaluated for the experimental aerodynamics standpoint. This field of application imposes requirements that have not yet been described in the literature. The experimental outcomes prove the surface quality does not suffice to conduct quantitative experiments. The holes that are necessary for pressure measurements in wind tunnel experiments cannot be reduced below 1 mm in diameter. The dimensional discrepancies are on the level beyond acceptable. Additionally, the problem of ‘reversed tolerance’, with the material building up and distorting the design, is visible in elements printed with the DLMS technology. The results indicate the necessity of post-machining of the printed elements prior their experimental usage, as their features in the ‘as fabricated’ state significantly disturb the flow conditions.

## 1. Introduction

The Direct Metal Laser Sintering (DLMS), known also as Selective Laser Melting (SLM), was developed by the EOS company and it has been commercially available since 1995 as the EOSINT M250 laser sintering machine. It is one of the additive manufacturing (AM) technologies. Unlike the traditional machining methods that rely on removing material, this one is based on building up the material onto the element that is being produced; a comprehensive review of AM is given by Bourell et al. in [1]. Briefly, the material is added as subsequent layers form a spatial element through such ‘quasi 2D’ layers. The shape of every layer corresponds to successive cross-section of the element to be produced and is generated by the CAM software, based on the CAD design. Defining the shape of each layer is referred to as the ‘slicing’ process. The layer thickness directly influences the precision of the shape produced; therefore, more advanced slicing algorithms adapt the layer thickness according to local value of curvature required to reduce the stepping effect in regions of high curvature. Such a process is explained by Yasa et al. in [2]. Nowadays, DLMS is a widely available technology implemented with metal powders. It employs a laser that directly illuminates the metal powder melting it and then a sintering process takes place in the liquid phase, thus forming a new layer. The laser scans the layer of raw material; after cooling down it forms a predefined layer of the designed shape. Each layer that is bond to the preceding one becomes solid by the time of sintering of the concurrent one.

As this technology enables producing three-dimensional parts directly from computer-aided design (CAD) software, it is very widely spread in many fields of science and industry. Depending on the requirements and applications, various types of metal powders or their alloys can be used in the DLMS printer: steel, nickel, cobalt, chrome, cooper, titanium, aluminium or tungsten, with the details given by the EOS company [3]. Some types of the powder require the sintering process to take place in a protective atmosphere of inert gas. The need for securing of the product from admixtures and by-products of oxidation process, especially in medical applications, is described by Raos et al. in [4].

The choice of the printing powder is dictated in the vast majority of cases by the required mechanical properties of the part to be printed. It was found that, as expected, the material used for printing impacts its mechanical properties, yet this is a complex influence. The properties of the stock, continuous material are different to the ones of the corresponding DLMS-produced element from the same material powder that was sintered. This phenomenon was examined by Gratton in [5]. Additionally, the ‘freshness’ of the powder is of significance. Opatová et al. analysed the properties of both new and re-used powder in [6]. They concluded that particles of much larger diameter are formed during the additive manufacturing process when reusing the powder. They also noticed a significant difference in the surface morphology of the new and re-used powder, favouring more homogenous layers obtained from the non-recycled material.

Becker et al. in [7] have proven that the tensile strength of the DLMS-processed element is influenced by residual stresses and microstructure cracks that are inevitable in the process, which involves rapid heating and cooling of the material. The authors of [8] analysed the influence of process parameters on the hardness of the DLMS-produced element. They came to a conclusion that hardness is influenced by scan spacing, followed by sintering speed and infiltration. Simchi [9] presented an attempt to describe the process analytically, looking into the densification and microstructural evolution during direct laser sintering of various metal powders. In conclusion, the author of the paper relates the empirical sintering rate to the energy input of the laser beam. He also proposes a model that includes operating parameters such as laser power, scan rate, layer thickness and scan line spacing.

Aside from the works on explaining the exact influence of the parameters governing the process on the final product, a lot of effort was also invested in optimizing of the values of the parameters. An interesting approach is given by Mierzejeska in [10]. Her paper describes an attempt to override the lack of full knowledge of the process parameters, mutual interactions and impact on the product by means of optimization using neural networks and genetic algorithms for this task. 

Due to the possible low quality of the outcome and tensile strength requirements, the printed element often requires undergoing further processing. Tan et al. [11] report significant improvement of strength after solution and aging treatment. Moreover, they succeeded in post-processing the grade 300 maraging steel produced by SLM additive manufacturing to the quality comparable to the standard wrought one.

Another parameter of interest for assessing the DLMS technology is the achievable geometrical precision of the printed element. Vranić et al. [12] gave some generally accepted guidelines for designing of parts. They suggest the minimal wall thickness of 0.5 mm or 1 mm, depending on the feature size. The authors assessed dimensional accuracy to be of 0.2% but not better than 200 µm of absolute size. They also recommended holes to be of diameter not smaller than 1 mm with space for re-drilling the hole to exact dimensions and suggests the achievable Ra of about 8 µm for a part ‘as fabricated’. The topic of possible diameter of holes in ‘as fabricated’ EOS printer maraging steel powder parts is described also by Dana et al. in [13,14]. The authors concluded their work by stating that holes of diameter between 0.5 mm and 10 mm can be successfully printed without supporting structures. Yet, for the small diameters, the quality of the hole is very limited. They notice that the maximum deviation of the circular shape is 0.3 mm, which is 60% for the 0.5 mm hole diameter. Additionally, the cross-sectional shape is far from circular for small diameters, being a result of the layers-like technology The holes of diameter below 0.5 mm are not investigated by the authors due to expected problems with removing the unprocessed powder after printing.

The SLS technology is still undergoing extensive improvement, which is summarized by Nagarajan et al. in [15]. The successing method, a micro-scale selective laser sintering (μ-SLS), is already available. Roy et al. in [16] describe the fabrication of three-dimensional parts with feature resolutions higher than 5 μm and a high throughput allowing it to be employed in a production environment.

The field of application of the DLMS technology is very wide, but the scope of this paper is its application to the production of turbine blades for wind tunnel experiments. The expectations for this method are very high. It is a result of the requirement to manufacture blades of complex geometry with internal cooling channels of very sophisticated shapes. Such objects are extremely difficult to machine. The technology of casting that is used for real-size blades is very expensive. The internal features (holes, channels) cause the moulds to be single-use. In this field of experiments, it has always been difficult to produce the correct geometry of the blade. The actual geometry of the blade is complex and originates from computational fluid dynamics. Since it is given as a set of coordinates over a rectangular mesh, it cannot be easily imported to a manual machining process. This problem is even greater as the size of wind tunnels is limited and it is often the case that the blade model for experiments needs to be reduced in size, making it even more challenging. The problem is significant, but the model blades can be produced by using a significant amount of work and a highly skilled craftsmen supported by an extensive machine park, as for it is described by Szwaba et al. in [17]. Such state-of-the-art technology does have a significant impact on the cost and time of preparing the experiment. Therefore, we have decided to investigate the possibility of using the SLM-produced blade without any post-machining or post-processing in the wind tunnel experiments.

## 2. Materials and Methods

The blade was designed to be a part of the experimental setup prepared at the IMP PAN, which is a supersonic wind tunnel facility. The wind tunnel is an intermittent, vacuum type wind tunnel. A detailed description is to be found in the work of Fomin et al. [18]; nonetheless, the crucial information is that the measurement chamber is 100 mm wide ‘spanwise’, which determines the maximum size of the blade that can be used for experiments. 

**Figure 1:**
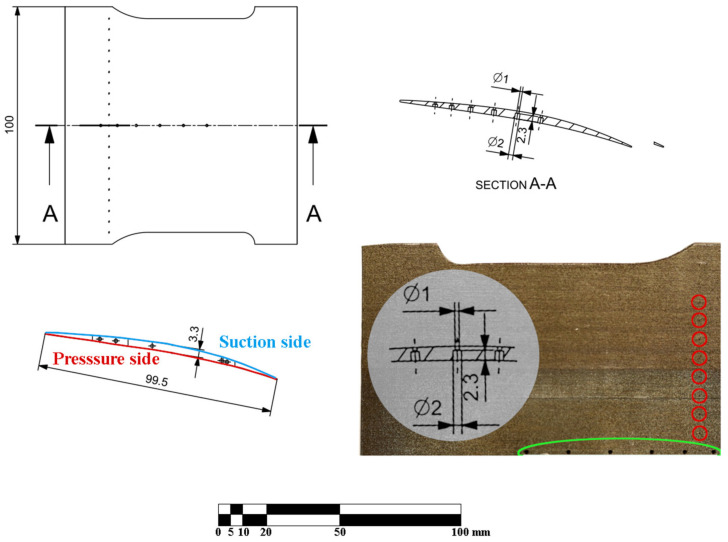
Blade geometry and the actual view of the printed element.

The geometry that was used as a model for printing is given in Figure 1.

The blade is close to being a square, with 100 mm spanwise and 99.5 mm chordwise in length. There are two rows of holes: one along the A-A cut and one close to the edge of the blade. One along the A-A cut is a counterbore hole changing its diameter from 1 mm to 2 mm at the depth of 2.3 mm from the suction side. For clarity, the geometry of these holes has been superimposed on the photograph of the blade in Figure 1 (the photograph with the imposed cross section is not-to-scale for readability). The latter row of holes is a set of 20 through-holes of 0.3 mm diameter. For clarity, the ‘pressure side’ and ‘suction side’ of the blade have also been marked with red and blue colour, respectively. The photograph in Figure 1 illustrates the SLM printed blade that was used in the experiments (it is the upper half, since the A-A plane is also the plane of symmetry for the blade).

The green loop indicates the position of the ‘large’ counterbore 1–2 mm holes and the red circles depict the position of every other of the ‘small’ 0.3 mm diameter holes. The blade was manufactured using an EOS M280 DLMS machine at IMP PAN KEZO Research Centre in Jabłonna, Poland. It is a powder bed fusion selective laser melting system equipped with a 400 W Yb fibre laser (wavelength of 1060–1100 nm). A printed part is manufactured by a laser exposure of sliced geometrical contours onto powder layers (typically of the height of tens of microns), which are applied from a powder dispenser chamber to a base plate by a recoater blade. As a result, the manufactured part is fully submerged in the metal powder. Selected material was EOS maraging steel MS1, providing excellent mechanical properties and straightforward post-treatment. The chemical composition of the alloy is given in Table 1 and corresponds to US 18% Ni maraging 300, European 1.2709 and German X3NiCoMoTi 18-9-5 steel defined in the corresponding datasheet [19].

The laser exposure was selected to be the ‘performance’ mode: a standard-setting provided by EOS, with a layer thickness of 40 µm. This choice results in a laser power of 258 W and a scanning velocity of 960 m/s. The detailed description of the exposure strategy can be found in previously published works of Kučerová et al. [20]. The manufactured elements without any post-heat treatment exhibit moderate anisotropy in terms of mechanical properties. Parameters for the material in ‘as-built’ condition are presented in Table 2.

**Figure 2:**
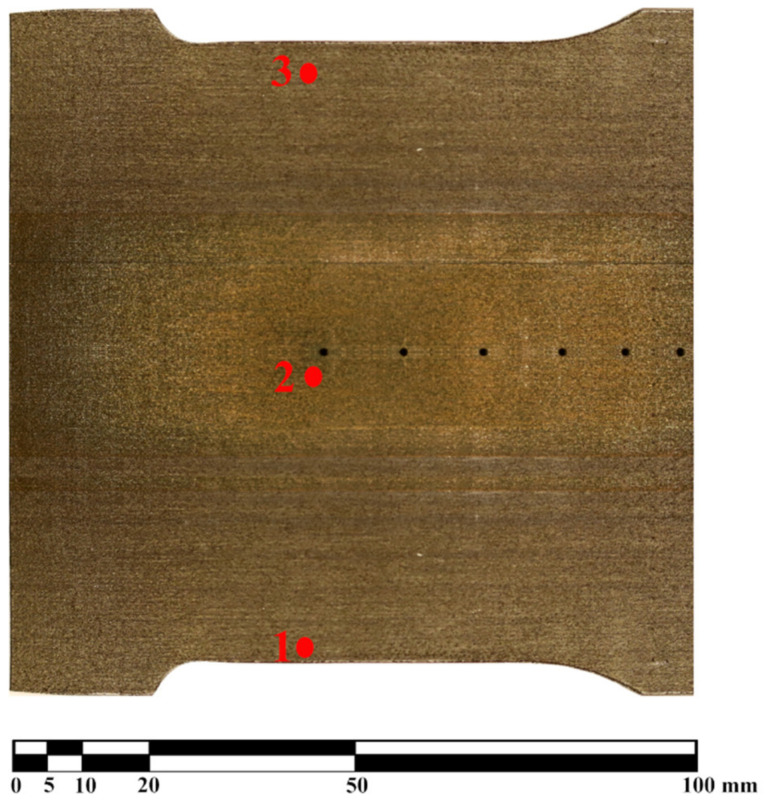
Positions of the roughness measurement points on the blade surface.

The parts were printed under the nitrogen atmosphere in the working chamber with oxygen content kept below 1.3%. The temperature of the base plate, on which the part is built, was kept at approximately 40 °C. No heat post-processing was applied after the printing. The part was positioned vertically. The principal axis of the profile was slightly inclined in the XY plane in order to provide better recoating and maintain high rigidity against the recoater arm. The positioning in the 3D space was a trade-off between the quality of the outer surface, quality of the holes and the support removal complexity. As the result, the layers are parallel to the A-A section plane defined in Figure 1, which is clearly visible in Figure 2 and all the following photographs.

The measurements that were performed included the assessment of:

- the quality of the surface (in terms of roughness),
- the quality of the 0.3 mm holes,
- the quality of the 1–2 mm holes,
- the precision of the overall shape.

All the roughness and hole quality measurements were conducted using the Nikon Eclipse Ti-S microscope and a dedicated software for measuring the surface profiles (roughness) and geometrical features (holes).

The roughness measurements were taken in six locations. They are marked with ‘1’, ‘2’, ‘3’ zones in Figure 2, and the other three are taken in the same locations on the other (pressure) side of the blade.

**Figure 3:**
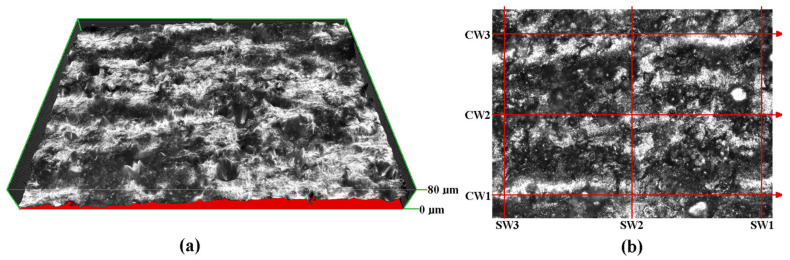
(**a**) Isometric projection of the surface profile. (**b**) The positions of the measured roughness profiles.

The numbers in Figure 2 indicate that the depicted blade was printed in the direction from the bottom (layer with point 1) to the top (layer with point 3). For each location of the three marked points there were measurements taken on both the ‘suction side’ and on the ‘pressure side’ of the blade. In every zone, six measurements were taken with three of them concerning the ‘spanwise’ roughness (SW1, SW2 and SW3) and ‘chordwise’ roughness (CW1, CW2 and CW3), in Figure 3b.

The distance between the ‘chordwise’ roughness profiles is 500 µm and the distance between ‘spanwise’ roughness profiles is 800 µm. The range of height in Figure 3b is 60 µm. For assessing of the quality of the holes, the measurements of geometry of the inlet and outlet and roughness of the interface between the two diameters of the hole were taken for the hole located on the ‘A-A section’ in Figure 1.

## 3. Results and Discussion

### 3.1. Roughness

The results describing the average roughness in terms of Ra value are given in Table 3. The Ra is defined as the arithmetical mean roughness value, being the arithmetical mean of the absolute values of the profile deviations from the mean line of the profile.

The values in Table 3 are averaged values of CW1, CW2 and CW3 or SW1, SW2 and SW3 for the given point. The values show that the very strong ‘visual’ anisotropy clearly visible in Figure 1 and in Figure 2, resulting from the orientation of the layers, does not have a significant impact on the value of Ra. This is in compliance with expectations based on mechanical properties given in Table 2. The material ‘as printed’ exhibits very low level of anisotropy and it turns out to correspond with the roughness. The corresponding values of ‘chordwise’ and ‘spanwise’ roughness are very similar. There is no clear relationship that was expected from the Figure 2; in all cases the ‘spanwise’ roughness is higher than the ‘chordwise’ one. This is caused by the size of the layers, as they increase the ‘waviness’, and the roughness is caused by the size of the powder particles that is not influenced by the direction of the layers. The table also proves that the values are in range corresponding to a coarse machining that is in agreement with the physical quality of the blade and available data [16,17]. The general Ra of the model is approximately 7 µm, corresponding to a very coarse machining. There is no dependence of the roughness, either on the curvature of the area or on the chronology of the printing process. This roughness is alike over the whole surface of the model that was investigated. This quality of the surface is a problem for any experiments concerning boundary layer, or experiments focusing on the spontaneous laminar-turbulent transition. Additionally, there is very clear directivity of the surface waviness (being a reflection of the layers) that makes it impossible to be used for oil visualization technique, since there is a very privileged direction that will influence the oil flow. Additionally, the value of Ra is too high for aerodynamic measurements, as even for unsophisticated aerodynamics, the Ra value should not exceed 1.0 µm.

### 3.2. Holes Quality

The ‘large’ 1–2 mm holes are marked with a green background and the positions of the ‘small’ 0.3 mm ones are marked with red circles in Figure 1. The quality of the ‘large’ holes has been measured in terms of the cross-section of the hole; for the ‘small’ holes these terms have turned out to be not applicable as measures of quality, as it turned out that the printing has failed to produce the ‘small’ holes feature.

#### 3.2.1. Quality of 0.3 mm Holes

**Figure 4:**
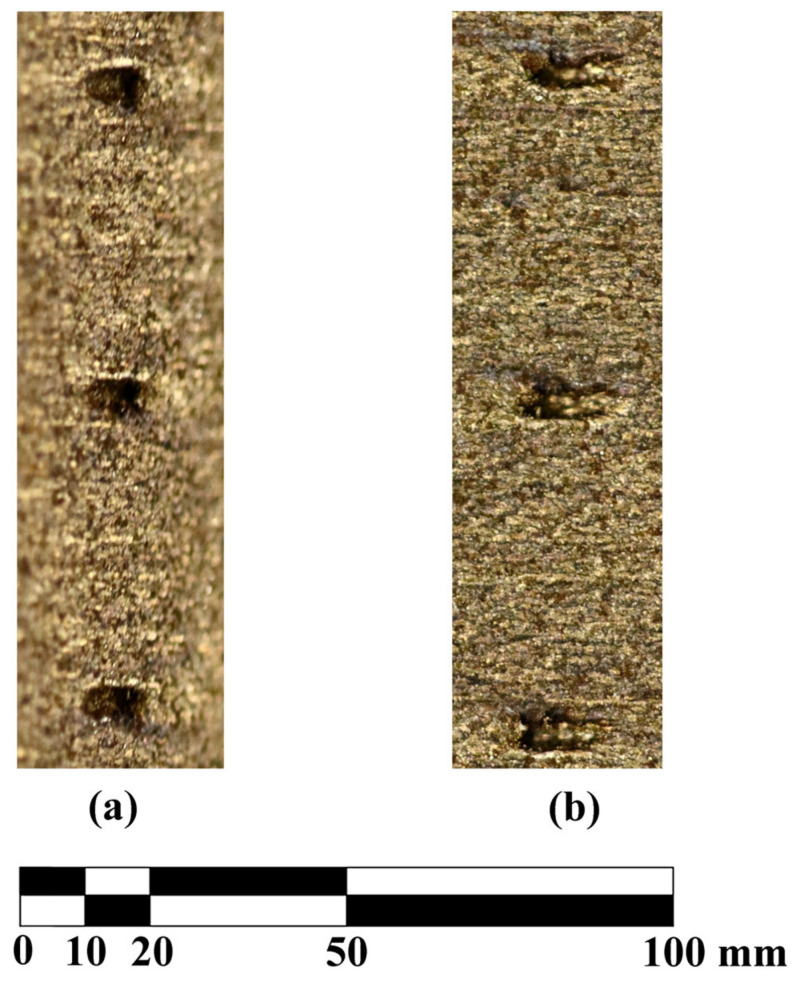
The detailed view showing the quality of the 0.3 mm holes in the printed blade. (**a**) The camera looking in the spanwise direction. (**b**) The camera looking in the cordwise direction.

The design diameter of the ‘small’ holes is 0.3 mm, which turned out to be too small for the printing process. An image showing three holes fabricated this way is given in Figure 4.

The image consists of two photographs showing the same holes. The left-hand side photograph is taken by camera rotated 90° clockwise compared to the viewing angle for the right-hand side photograph. This enables visualisation of the spatial distribution of the feature. The positions of the holes can be distinguished by some visible artefacts. The internal part of the features is filled up with the material that is uniformly melted and there is no passage through the blade. Tested permeability of these features yielded zero for gases; hence the conclusion they are tightly filled with a solid material. The permeability has been measured in a macroscopic sense that is appropriate for the aerodynamic research we focus on. The blade (with counterbore holes locked) has been exposed to differential pressure of 1 atm (corresponding to expansion to vacuum that is the ‘ideal’, extreme case for supersonic flows) and no through flow has been detected.

#### 3.2.2. Quality of 1–2 mm Holes

**Figure 5:**
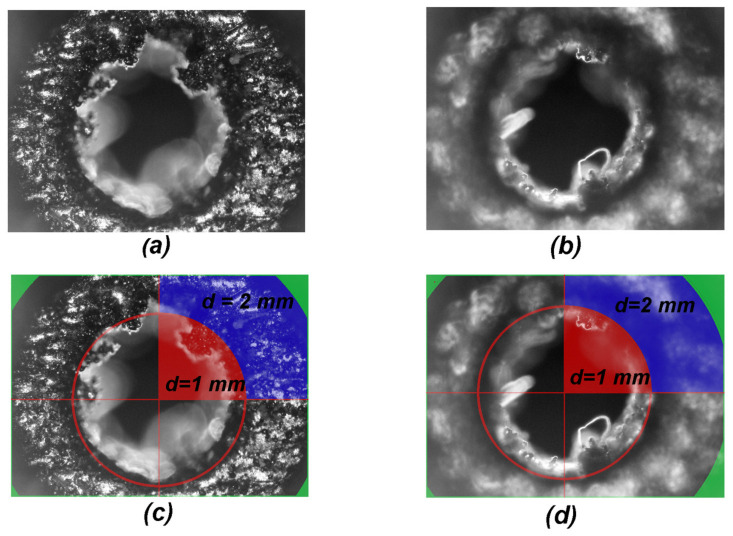
Microscopic images of the ‘large’ hole. (**a**) The focus on the 2 mm part of the hole, (**c**) quantification of the 2 mm part of the hole, (**b**) the focus on the 1 mm part of the hole and (**d**) quantification of the 1 mm part of the hole.

As mentioned before, the design diameter of the ‘large’ holes changes from 2.0 mm on the pressure side to 1.0 mm at the suction side. Two microscopic photographs of such hole, as viewed from the pressure side, are shown in Figure 5.

Both photographs in Figure 5 were taken from the pressure side of the blade; they only differ by the position of the focal plane adjustment. The (a) image is focused on the plane, where the diameter of the hole changes; the (b) image is focused approximately 0.5 mm below, halfway the length of the 1 mm diameter part of the hole. The photographs (c) and (d) have been augmented with dimensions and auxiliary shapes. The photograph (c) is augmented photograph (a) and photograph (b) is the raw material for photograph (d). The single red circle marks 500 µm of radius that corresponds to the designed 1 mm diameter hole. The radius of the edge of the solid blue region is 1000 µm, corresponding to the designed 2 mm diameter hole. The solid red area in the first quadrant illustrates an ideal hole of 1 mm diameter, while the zone restricted by solid blue corresponds to exact 2 mm hole.

In Figure 5, the panel (a) a solid blue sector in the first quadrant depicts the correct area of the plane of changing the diameter. It is clearly seen that neither the external nor the internal diameter is conserved. The zone is reduced by approximately 150 µm on the external radius; hence, as seen in the second quadrant, the actual diameter is not 2 mm as designed, but only approximately 1.7 mm. Additionally, the inner radius of this region is reduced by a similar value, and thus the solid material is vastly seen in the area surrounded by red line, reducing the cross section of the internal hole.

From the image in panel (b) the quality of the 1 mm part of the hole was assessed. The focal plane is about 0.5 mm inside the hole that was designed to be 1 mm in diameter. The red sector in the first quadrant illustrates the desired area of this aperture that should be free of any residual material within the red solid line circle that marks it. Again, the diameter of the hole is reduced on average by approximately 150 µm, but there is debris reaching the radius of 200 µm, thus reducing the radius of this opening by 300 µm, which is 60%.

Both the radii are internal, and the reduction is caused by the principle of the LMS operation. The path of the laser beam may be assumed to be correctly following the desired geometry of the element, yet the melting process takes place in a zone of finite dimensions. Additionally, as the material is heated up, the heat propagates to its surroundings, causing the adjacent dusting of the material to melt and build up an excessive volume that reduces the apertures. The diameter of the designed holes is reduced even by approximately 300 µm. For the typical 1 mm or 2 mm diameter holes that are used for pressure taps in wind-tunnels, this is about 20% reduction of the original diameter. The reason for that results from the material being heated up and the heat propagating to the surrounding material. The presence of heat outside the assumed geometry causes the adjacent material to build up an excessive volume on the surface of the generated aperture, resulting in the reduction of the geometry of the hole as compared to the original design. It also made it impossible to print the holes of 0.3 mm diameter. Therefore, the limit for the diameter of the hole that can be produced with presented DLMS setup is between 0.3 mm and 1.0 mm. This restriction is a problem for blades with channels for flow control that have to be re-drilled.

### 3.3. Shape Dimensional Precision

**Figure 6:**
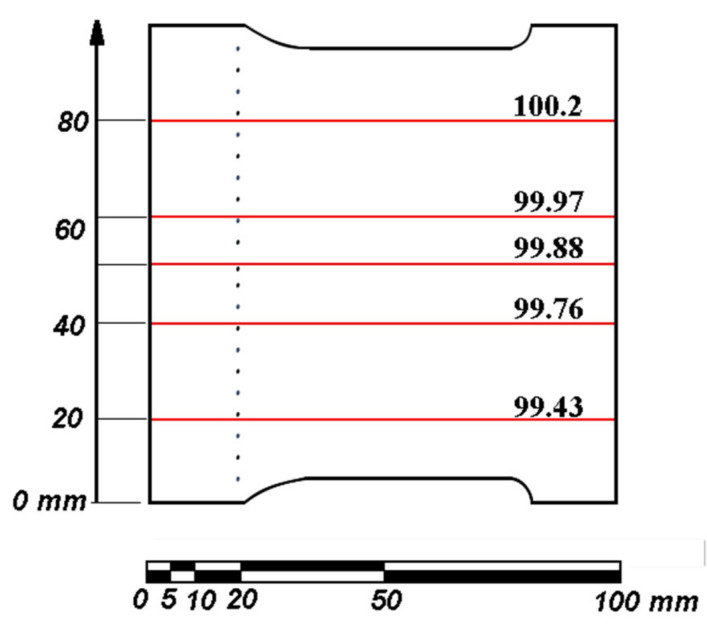
The actual chordwise dimensions of the printed blade.

The measured values of the external, macroscopic dimensions are marked as red lines in Figure 6. The vertical positions of measurement lines are marked on the auxiliary system of coordinates with the zero value corresponding to the bottom surface of the blade.

The blade design, given in Figure 1, defines the chordwise dimension as 97.7 mm, whereas the physical, manufactured dimensions were obtained approximately 3% larger. It is also worth noticing that the absolute error in this dimension is clearly rising as one moves along the span of the blade, i.e., vertically in Figure 6. The first layer that was produced is the bottom one in Figure 6, and the process proceeded upwards; hence, the amount of the excessive material that was being incorporated into the model was increasing as the time of the process elapsed. This may be caused by heating up the model and the whole pile of the powder that had not yet been melted. The dimensions were increased by the mechanism that has been described when assessing the holes’ quality. It is also worth mentioning that, for large features, the magnitude of this error is a function of the time (as counted from starting the printing) of melting to the point that is being considered. This is a result of accumulation of the energy in the model and the heap of metal powder during the printing. This is a serious concern when considering supersonic flows in wind tunnels. Changing the cross section of the blade in supersonic flow influences the local Mach number and conditions in a significant manner. As this change is variant along the span, it also makes it impossible to use optical methods of visualisation such as the very widely used basic schlieren described by Smits in [21] visualization, or the FTFA interferometric analysis described by Telega et al. in [22], since there the result is ‘integrated’ along the optical path, i.e., across the tunnel.

## 4. Conclusions

This paper examined features of the DLMS-produced blade model that are crucial when assessing its applicability in aerodynamic research that always includes equipping the blade model with holes for pressure measurement tabs and aerodynamically smooth surfaces for various visualization techniques. It is clear that the ‘as fabricated’ blade cannot be used in wind tunnels without post-machining. The dimensional discrepancies require finishing milling in order for the blade to fit into the bed at the measurement stand. The surface quality in terms of average roughness is not sufficient as it influences the flow in terms of boundary layer and heat transfer coefficient. The holes necessary for pressure measurement cannot be manufactured to be smaller than 1 mm diameter with this method.

Due to the above reasons, the DLMS-printed blade does require fine finishing by means of ‘classical’ mechanical machining prior to use in wind tunnel tests, especially as there is excessive material that is causing ‘reversed’ tolerances. In the range of cross sections typical for turbine blade models in supersonic wind-tunnels, if the cross sectional area of the hole is of importance for the flow, they have to be re-machined to the correct geometry as the dimensional error of 200 µm is already significant. Hence, using the ‘raw’ DLMS-printed parts is not possible in the experimental aerodynamics without post-machining. Producing models for wind-tunnel tests, being aware of the necessity of fine-machining and taking into account that it is a one-off production, it can still be beneficial when compared to ‘classical’ methods in terms of cost and time. For more complicated shapes, including steep edges and thin elements, the requirements are becoming more challenging. In the case of big models, low flow velocities and specific cases, the problems might be less significant, but the assessment needs very careful consideration.

## Figures and Tables

**Table 1:** Mass chemical composition of maraging steel MS1 [19].

Fe	Ni	Co	Mo	Ti	Al	Cr, Cu	C	Mn, Si	P, S
balance	17–19	8.5–9.5	4.5–5.2	0.6–0.8	0.05–0.15	<0.5	<0.03	<0.1	<0.01

**Table 2:** Mechanical properties of maraging steel MS1 in ‘as-built condition’ [19].

	Direction	
	Horizontal XY	Vertical Z
tensile strength	1100 ± 100 MPa	1100 ± 100 MPa
yield strength 0.2%	1050 ± 100 MPa	1000 ± 100 MPa
elongation at break	typ. 10 +/− 4%	typ. 10 +/− 4%
elasticity modulus	typ. 160 ± 25 GPa	typ. 160 ± 25 GPa
hardness	typ. 33–37 HRC	

**Table 3:** Roughness average—Ra values.

Side	Direction	Point	Ra
			µm
Pressure	Cordwise	1	9.38
		2	5.45
		3	5.21
	Spanwise	1	8.4
		2	7.42
		3	4.91
Suction	Cordwise	1	5.61
		2	6.37
		3	5.92
	Spanwise	1	7.51
		2	5.12
		3	4.01

## Data Availability

Data sharing is not applicable to this article.
